# Nursing in Workhouse Infirmaries

**Published:** 1887-03-26

**Authors:** 


### Nursing in Workhouse Infirmaries.

At the last meeting of the Hospitals Association, the attendance included the following: Miss Davenport Hill (Paddington Guardians), Mrs. Bluett and Miss Walters (Fitzroy House), Miss Wilson (Secretary of the Workhouse Infirmary Nursing Association), Mrs. Peake, Miss Hendon, Miss Manning (of St. Thomas's Hospital), Miss Venables (of St. Thomas's Hospital), Miss Griffiths (Matron of Lambeth Infirmary), Miss Haddock (Matron of the City of London Infirmary), Miss Manson and Miss Turner (of St. Bartholomew's), Miss Parsons (Chelsea Guardians), Miss W. Parsons, Mr. Millward (Clerk to the St. Pancras Guardians), Dr. Adams (Chairman Highgate Infirmary), Dr. Stretch-Dowse, Mrs. O'Rell (St. Bartholomew's), Miss Walter, Miss Nichols, Miss March, Miss Franks, Miss Coghlan, Miss M. Webster (St. Thomas's Hospital), Miss Wood, Mrs. Burdett, Mrs. Perry (Matron of the Whitechapel Infirmary), and others. The chair was taken by Dr. Bristowe, Senior Physician at St. Thomas's. The following discussion took place upon the paper.

Col. Montefiore, in thanking Mr. Benton for his very useful paper, said that there was not the slightest doubt that, if matrons were always ladies trained to nurse the sick, it would not only be very much better for the sick poor, but also for the whole tone of the conduct of infirmaries. There were some infirmaries where they had these ladies who were trained, and one could at once see in these infirmaries the extraordinary difference in the tone. At two infirmaries which he had the pleasure of going over last year, the difference was most marked. Formerly, when he paid his first visit to one of these infirmaries, they had a matron who was not a lady, and had not been trained. She and the matron at the other infirmary to which he referred, were only there as housekeepers. On his second visit the whole condition of things had changed. There would, he thought, be very few people found who did not think that trained ladies should be got into all Poor-law Infirmaries. There must of course be some difficulties in the way. There might be women who had served well in some of these infirmaries. The Guardians had no other position to which they could transfer them, and they did not like the idea of pensioning them off. The question of adequate accommodation for the nurses was gaining ground. At the new Dulwich Infirmary very admirable quarters had been built for the nurses. He was sure that all the medical superintendents he had seen would be only too glad if they could obtain a still higher class of nurses for their infirmaries. As the writer of the paper very justly said, the medical staff was so much overworked, that unless they had a very high class of nurses who did not want to be always overlooked, the sick poor would suffer. The doctors had to pay their attention to the more acute cases, and unless the nurses on their own responsibility paid attention to the general cases, the sick poor would suffer very considerably.

Dr. Adams quite agreed with all that had fallen from the last speaker in regard to the appointment of matrons and nurses to our infirmaries. He certainly thought that the matron should be a lady; moreover, he agreed with the writer of the paper that she should be a trained nurse as well, so that she might exercise her influence over the nursing staff, as well as over the patients. He would say nothing more in regard to the nursing, for it had been very ably dealt with in the paper. He would, however, say something in regard to the opening of infirmaries for the purposes of clinical instruction. He thought Mr. Benton was mistaken in supposing that Mr. Gathorne Hardy's Act interfered with the usefulness of these infirmaries for medical instruction. His Act did the very opposite, for it gave the right of opening these infirmaries for medical instruction, but subsequently the particular clause which gave that power—the 29th clause—was repealed, and so these infirmaries had from that time been shut against the medical profession and clinical instruction for young medical men. He took a good deal of interest in the infirmary of the parish of St. Pancras. His chief object in seeking a seat at the Board of Guardians of that parish was with a view to opening up the infirmary to the medical profession, not as a medical school, but as a place for clinical experience for young medical men, for that was greatly wanted by the medical profession. By so doing, a great number of medical men of the highest class would be attracted, who would work and study within the infirmary, and thus more knowledge must be brought to bear on the treatment of difficult diseases. Thus, not only would the poor benefit by the opportunity of such treatment, but likewise the ratepayers by the shortening of the duration of the stay of the sick poor in the infirmaries. All this had been very successfully accomplished, and he thought that St. Pancras had given an example to all England in opening the wards of her infirmary for medical instruction. At St. Pancras they had now ten probationers in the infirmary who were paid at the rate of £10 a year, and served one year's probation, after which they took up office where the guardians chose to send them. They received instruction under the medical superintendent and the matron, and were taught physiology, anatomy, etc., and the general duties of nurse. They were a superior class of young women, and they mostly turned out very efficient nurses. He did not agree with Mr. Benton that they should perform the duties of night nursing. The night nurses were placed in quite as responsible a position as the day nurses. This had been recognised at his infirmary by the appointment of trained nurses for the night work. To place a probationer in the sole charge of a ward was, he considered, placing her in a position which ought not to be thrust upon her.

Dr. T. Stretch-Dowse observed that he knew no man who had more experience, who had given more attention to the subject, and was better able to draw up a paper of this kind than Mr. Benton. Mr. Benton was his colleague at the Highgate Infirmary for some years, where they had worked together hand in hand to promote the welfare of the institution. When he (Dr. Dowse) joined the infirmary, they had an exceedingly well-trained class of nurses from St. Thomas's Hospital. Indeed, he thought they began under most favourable auspices, and it was generally regarded by the Poor-law Board and others who visited the institution, that it was worked as well as such an institution could possibly be worked. At its head they had a trained nurse—a lady—and the other ladies under her received their training under the Nightingale system adopted at St. Thomas's Hospital. At one time the system rather fell through. During the time he was there, he and Mr. Benton gave lectures to the nurses, and did their best to keep them in training. However, it did, as he said, fall through, and it was certainly thought that the nursing system at Highgate, if not perhaps demoralised, was certainly anything but perfect. He merely alluded to this fact to bear out the remarks of his friend, Mr. Benton, that it was highly necessary to have a trained matron and a lady. Before sitting down he would like to say a few words in reference to workhouse infirmaries in their character as State hospitals. He was very pleased to hear that his old infirmary workhouse was the pioneer in this direction. The fact that they had two clinical clerks there was quite sufficient to lead them to the opinion that they had applied the thin end of the wedge, and that by-and-bye they would have in the wedge itself. He felt perfectly certain that it was merely a question of time. He should be happy to see the day when they were State institutions, and no longer governed by Boards of Guardians, unless they were all guardians like Mr. Adams. (Hear, hear.) There was a vast field of work in London for the medical students at these workhouse infirmaries. (Hear, hear.) There could be no question about it at all. If they have lady doctors—to whom he had no objection, for they might ornament the profession—let them have this field of work. (Hear, hear, from a lady.) He congratulated Mr. Benton on having brought forward these points in a very clear, simple, and concise way.

Miss Wilson, the honorary secretary of the Workhouse Infirmary Nursing Association, said she should be pleased to hear some discussion of the question of the possibility of maternity training being provided in infirmaries. It was a very pressing question : trained midwives were wanted to supply the country quite as much as the town, and there was an absolute need that the nurses sent to the country should be certificated midwives. In this matter there was a very fine experience to be had in our infirmaries, but at present there were exceedingly few openings. She particularly desired to urge that the maternity wards in the London infirmaries should be open. She should be pleased to hear what practical difficulties there were in the way.

Miss Davenport Hill said that at St. Pancras they had a sort of half infirmary department with a staff of nurses, but a very large proportion of the cases were chronic and uninteresting, and therefore the workhouse was not attractive. She supposed the Guardians of other parishes had the same difficulty in getting quite the right class of nurses because of this absence of strong interest. There should be different ways of making life attractive for the nurses; for instance, the dietary should be very carefully attended to. This was a most important matter, for, unless the nurses were comfortably fed, they could not keep up their health under work which was certainly depressing.

Miss Wood referred to the number of ladies who were seeking for employment at the present time. She had to refuse some half-dozen applications per diem from ladies who applied to her for nursing work. She wished to ask if it would not be possible for the Local Government Board or the Boards of Guardians to find employment for these ladies. They would, she was sure, be willing enough to go and learn nursing if only they knew that they were wanted. There would be a large supply of such women, if only the means could be found by which the two parties—those who wanted nursing and those who wanted employment—could be brought together. The bedroom accommodation for nurses at many workhouses was unsatisfactory. The least that any woman could ask was that she should have a separate cubicle, and that her meals should be comfortably served. If the sleeping accommodation was good, and the food was well served, there were many ladies who would come forward. While there need be no extravagance, a wide field of employment would be opened to ladies who were obliged to earn their own living.

Miss Griffiths endorsed what Miss Wood had said, and detailed the improvements which she had been able to accomplish in the matter of the nurses' dietary at the Lambeth Infirmary.

Mr. Millward, clerk to the Guardians of St. Pancras, said that at some institutions there was a repugnance among the nurses to dining in a body. They preferred to have their meals in their own rooms; it was so at his institution.

Mr. Benton, replying, said he was much obliged to them all, especially to the ladies who had taken part in the discussion. He was especially pleased to hear that St. Pancras was taking the lead in infirmary work. In respect to what Dr. Dowse had said about the probationer being on night duty, he quite agreed with him. Dr. Adams, he thought, had misunderstood his remark What he stated was, that when the probationer was out of her training, and was probably qualified, she should, before being given the authority of a ward, be given some night-work. Miss Wilson very properly laid great stress upon the question of maternity training. He hoped Guardians would allow the midwifery wards to be thrown open for training nurses. He thought all nurses should be made to dine in the hall, unless they got special permission from the matron. Nurses who were very friendly might be put at separate tables. (Laughter.)

Dr. Bristowe, the Chairman, read a letter from Mr. Lunn, the Medical Superintendent of the Marylebone Infirmary, who was unable to be present. The subject they had first discussed was one which interested him very greatly. Nurses, he considered, required good treatment and exceptional kindness, for their duties were very exacting. In every little detail they should be looked after, and they should be comfortably housed at night. Great care should be devoted to their dietary; it was a matter about which there ought not to be any very great difficulty. He recollected the time when at the London hospitals the nursing was on a most abominable system. The nurses—all except some of the higher ones—used to live outside the hospital and provide their own food. There was nothing more abominable than that mode of procedure. At St. Thomas's the nurses now dined together, and the plan worked exceedingly well, and should be adopted at all hospitals and infirmaries. Miss Davenport Hill had referred to the dreariness which nurses had to endure in workhouse infirmaries. In workhouses where the patients were mostly old and remained long, the work must necessarily be dreary, and therefore, if they wished to secure the services of good nurses, they must give them some amusement. There was no doubt that nursing was an employment for ladies which was largely on the increase. Infirmaries were, he considered, very suitable places for the instruction of nurses. The midwifery training, to which Miss Wilson had referred, should form a most important part in the instruction of nurses at the infirmaries. The matrons of infirmaries should be ladies—ladies of breeding. There were many ladies at the present time who wanted employment, and were anxious to become nurses, and found a difficulty in obtaining instruction. At St. Thomas's a very large number of ladies applied for places as probationers, but they had no room. He approved of the utilisation of infirmaries for medical teaching, and he believed that in the future they would be the medical schools of the country. He thanked Mr. Benton for his excellent paper.

Mr. Burdett announced that the next meeting of the Hospitals Association would be held on the 20th April, at the Brompton Hospital for Consumption. This was a new departure, but a wise one. He believed that it would add to the interest of the discussions and papers on practical questions if the meetings were held in the board-rooms of the hospitals. Dr. Williams, one of the staff, would give a most interesting paper on a subject intimately connected with the treatment of phthisis, and there would, no doubt, be an opportunity for seeing the new buildings at Brompton. He read a letter from Miss Louisa Twining, who had done more for the cause of workhouse reform than probably anybody else in this country.

---

We may state that at the St. Pancras Infirmary wing of the workhouse, a common room, containing a library of interesting books, has been established, chiefly through the influence of Miss Davenport Hill and other lady guardians, for the recreation of the nurses in their leisure hours.

